# Manufacturing of Ultra-Thin X-ray-Compatible COC Microfluidic Devices for Optimal In Situ Macromolecular Crystallography Experiments

**DOI:** 10.3390/mi13081365

**Published:** 2022-08-22

**Authors:** Ramakrishna Vasireddi, Antonin Gardais, Leonard M. G. Chavas

**Affiliations:** 1 Synchrotron SOLEIL, L’Orme des Merisier, Saint-Aubin, 91192 Gif-sur-Yvette, France; 2 Department of Applied Physics, Nagoya University, Nagoya 464-8603, Japan; 3 Synchrotron Radiation Research Center, Nagoya University, Nagoya 464-8603, Japan

**Keywords:** COC, device fabrication, diffusion, room temperature data collection, serial crystallography

## Abstract

Cyclic-olefin-copolymer (COC)-based microfluidic devices are increasingly becoming the center of highly valuable research for in situ X-ray measurements due to their compatibility with X-rays, biological compounds, chemical resistance, optical properties, low cost, and simplified handling. COC microfluidic devices present potential solutions to challenging biological applications such as protein binding, folding, nucleation, growth kinetics, and structural changes. In recent years, the techniques applied to manufacturing and handling these devices have capitalized on enormous progress toward small-scale sample probing. Here, we describe the new and innovative design aspects, fabrication, and experimental implementation of low-cost and micron-sized X-ray-compatible microfluidic sample environments that address diffusion-based crystal formation for crystallographic characterization. The devices appear fully compatible with crystal growth and subsequent X-ray diffraction experiments, resulting in remarkably low background data recording. The results highlighted in this research demonstrate how the engineered microfluidic devices allow the recording of accurate crystallographic data at room temperature and structure determination at high resolution.

## 1. Introduction

The design, choice of material, and fabrication methods are important factors to be considered in the manufacturing of microfluidic devices (MiDs), more specifically when applied to X-ray experiments [1,2,3,4,5]. The rapid fabrication and low cost of the material need to be suitable for processing and fabricating modern MiDs that allow for the arrangement of microchannel geometries as integrated using computer-aided design (CAD) tools [6,7]. In contrast, these materials are proven to have chemical resistance and good mechanical properties to withstand the applied pressures for handling the fluids. The above requirements are generally mandatory before the bonding, surface treatments/modifications, and sealing steps for microchannels [8,9].

MiDs are fabricated from various advanced polymer materials used either individually or in a combination in soft lithographic replication moldings. A non-exhaustive list of the most-utilized polymers includes polydimethylsiloxane (PDMS) [10,11,12], Norland Optical Adhesive 81 (NOA81) [13], OrmoComp^®^ [14], hot embossing (e.g., perfluoroalkoxy-PFA) and cyclic olefin copolymers (COCs) [15], and laser structuring (Kapton) [16]. With the advent of more powerful and brighter X-ray sources, Kapton and COC-based devices have received tremendous interest when integrated within sample environments due to their X-ray compatibility and low background noise [2,5,17]. A manifest drawback of Kapton or Kapton-metal devices lies in their difficult handling and fabrication procedures, which prevents potential rapid prototyping. COC-based devices, on the other hand, combine both desired qualities: X-ray compatibility and rapid prototyping [18]. To avoid compromising the advantageously low background properties of COC, the addition to the devices of materials opaque to X-rays should be avoided. Furthermore, its qualities as a thermoplastic make COC a simple element to handle for hot-embossing and injection molding of microfluidic channels [1,4,19,20].

In macromolecular crystallography, the X-ray diffraction experiments consist of measuring the intensities diffracted by a sample exposed to an X-ray beam. The detection and measurement of these intensities are strongly dependent on the background noise generated by diffusing elements crossed by the incoming X-ray beam. In this context and to optimize MX experiments, the microfluidic devices need to be adapted to avoid adding unnecessary noise to the experiments, which tends to be a difficult task because of the X-ray absorption of most of the device materials used to make the devices [5,21]. In this ongoing transfer process, a variety of fabrication approaches and multiple types of devices have been developed [5,22,23]. Currently, the combination of state-of-the-art instrumentation to adapt microfluidic-based sample-handling devices within experimental environments at microfocusing X-ray end-stations from synchrotron and X-ray free-electron laser sources is massively pursued, notably to gain further insights into the structural dynamics of biomolecules [8,24,25,26,27]. In the current investigation, we developed X-ray-compatible MiDs with highly reproducible geometric features suitable for diffusion-based crystal formation for X-ray diffraction experiments at synchrotron sources [2,27,28]. The devices were inspired by previously reported developments, however providing the experimenter with an optimized design that reduces the experimental background caused by the materials composing the MiD. To the best of our knowledge, the MiD introduced in this work presents features of thin layers of COC that are unprecedented in macromolecular crystallography, ideal when experimenting at hard X-ray synchrotron facilities. High-quality X-ray diffraction data were collected on protein crystals grown directly within the chip, with an exceptionally low background recorded on the images, a clear advantage of the devices while performing in situ macromolecular crystallography experiments.

## 2. Materials and Methods

**Figure 1:**
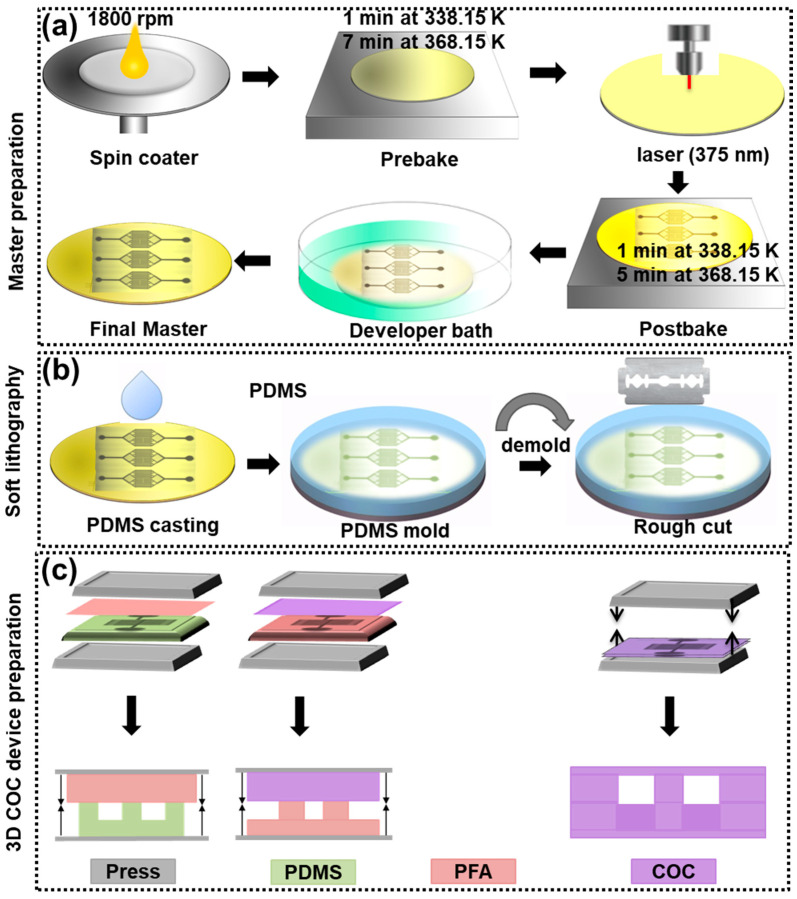
Combination of soft lithography and hot-embossing techniques for COC microfluidic device fabrication. (**a**) The photolithographic master fabrication involves spin coating, baking, and engraving with a laser writer. (**b**) After development, the uncured photoresist is removed and the resulting microchannel is replicated using polydimethylsiloxane. (**c**) The replica is peeled off the master device, which is used as the template for the PFA-intermediate stamp, which transfers the structure onto the COC.

The COC devices were fabricated in multiple steps. A photoresist master was produced using UV-lithography. The master was used to prepare a PDMS stamp applying soft lithography, which was then employed to hot-emboss the PFA polymer. This PFA-stamp was in turn utilized to hot emboss the COC polymer as a mold that could be bonded to obtain the final sealed devices (Figure 1).

### 2.1. Photo-Lithographic Master Fabrication

The master mold fabrication process (Figure 1a) was initiated by spin coating a 3″ silicon wafer with a negative photoresist (SU-8 2050, at 2500 rpm, Microchem Co., Saint-Rémy-de-Provence, France). After pre-backing the coated layer for 1 min at 65 °C followed by 7 min at 95 °C, the inverse structures were engraved using a laser writer (KLOE-Dilase 250), with structures previously drawn through some initial CAD design. The structures were again baked stepwise for 1 min at 65 °C and 5 min at 95 °C, before development under propylene glycol monomethyl ether acetate (PGMA).

### 2.2. COC Device Fabrication

The SU-8 masters fabricated above were used to transfer their inverse channel structures into PDMS molds by soft lithography (Figure 1b). The PDMS (Sylgard 184 kit, Dow corning CO, Midland, MI, USA) was mixed with a curing agent in a 10:1 ratio, poured onto the SU-8 master placed in a Petri dish covered with aluminum foil to prepare a thin PDMS mold. The mixture was degassed in a desiccator to remove all air bubbles before baking at 75 °C for 2 h. The cured PDMS mold could then be peeled off the master.

The PDMS molds were further used as templates for fabricating perfluoroalkoxy (PFA) stamps via hot embossing. PFA polymer granulates (grade PFA FLEX 8515UHPZ-Dyneon GmbH, Burgkirchen an der Alz, Germany) were hot-pressed to PFA sheets of ~500 μm using a LABOPRESS P150H hot press. The PFA was centered in a metal frame sandwiched by two Kapton foils in the preheated hot press and cured for 5 min. The hot plates were heated at 300 °C and pressed together to reach a force of ca. 0.6 kN for 20 s, allowing the PFA to melt slowly before increasing the force to 10 kN, maintained for 10 s. Next, the pressure was released down to a force of 5 kN slowly before being increased again to 10 kN. After reaching 10 kN, the system was maintained stable until the end of the 5 min overall process. The polymer was actively cooled, and the finished sheet was removed at room temperature. A similar protocol was applied to prepare COC polymer granulates (grade COC-8007X10-Dyneon GmbH) into sheets of ~700 μm, with small variations regarding the force (ca. 6 kN instead of 10 kN) and the hot plate temperature (160 °C instead of 300 °C).

The PDMS mold with the micro-structured channel was pre-baked for 15 min in between two Kapton foils at low forces (0.1 kN) and 200 °C. After releasing the force, a piece of PFA sheet was placed on top of the baked PDMS stamp, here again, sandwiched by two Kapton foils and placed into the hot press for hot embossing. The upper plate of the press was heated to 325 °C, while the lower plate was kept at 200 °C. The curing time and force were set to 7 min and 0.3 kN, respectively. After curing, both plates were cooled to room temperature while keeping the stack under pressure. Subsequently, the inverted structured PFA sheet was hot-pressed into the 700 µm-thick COC sheet, the COC sheet placed onto the PFA stamp, and the upper and lower plates both heated to 220 °C for 10 min at a force of ca. 0.2 kN. The plates were then left to cool to room temperature before removing the structured COC sheet (Figure 1c). Inlet and outlet holes (0.8 mm) were drilled, and the sheets were prepared for solvent bonding. The bonding step followed a previously reported procedure [9], where the two halves of the COC device were chemically sealed before being placed on the PDMS, sandwiched between two Kapton foils, and pressurized at a force of 0.9 kN and a temperature of 65 °C for 8 min. The final device was then actively cooled to room temperature.

### 2.3. Sample Loading and Crystallization

**Figure 2:**
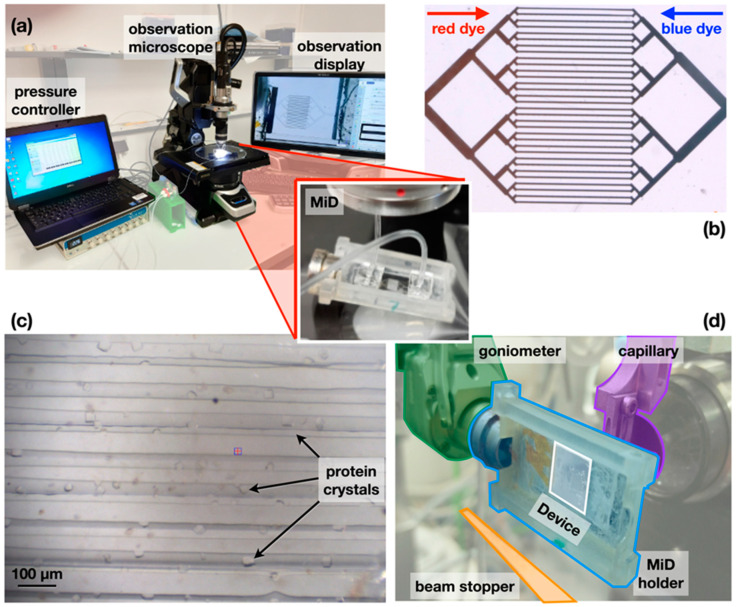
Overview of the workflow implemented at Synchrotron SOLEIL for developing and using the MiDs. (**a**) Injection sample for loading crystallization and protein solutions in the chip with careful inspection under a digital microscope. (**b**) Confirmation of the injection protocol using two colored solutions. (**c**) Appearance of protein crystals inside the channels after 14 h growth at room temperature. (**d**) Handling of the MiD and its holder at the experimental station of the PROXIMA-1 beamline for collection of in situ serial crystallography diffraction data.

The sample was injected into the MiD using tubing (LDPE micro medical tubing, Scientific Commodities; inner and outer diameter of 0.38 mm and 1.09 mm, respectively) connected to the inlet and outlet of the chip and on a mass flow controller for easier control of the injection (Figure 2a). The injection process was confirmed by injecting two colored dyes (red E129 and blue E133, Mallard Ferriere, Noisy-le-Sec, France) to mimic the precipitant and protein solutions (Figure 2b), respectively, with the observation of the mixing under a digital microscope (VHX Keyence, Osaka, Japan). After validation of the injection protocol, HEWL Lysozyme (Sigma-Aldrich, Saint Loius, MO, USA) crystals were grown inside the chip by diffusion after injecting the protein solution (~10 mg/mL) in the chip previously loaded with the crystallization solution (1 M NaCl, 35% *w*/*v* Ethylene Glycol, 12.5% *w/v* PolyEthylene Glycol-3350, 50 mM NaOAc/HOAc). The crystals of the protein grew after 14 h with dimensions distributed around the size of the channels, between 20 and 50 microns in the largest dimension (Figure 2c).

### 2.4. Data Acquisition, Processing, and Structure Determination

**Figure 3:**
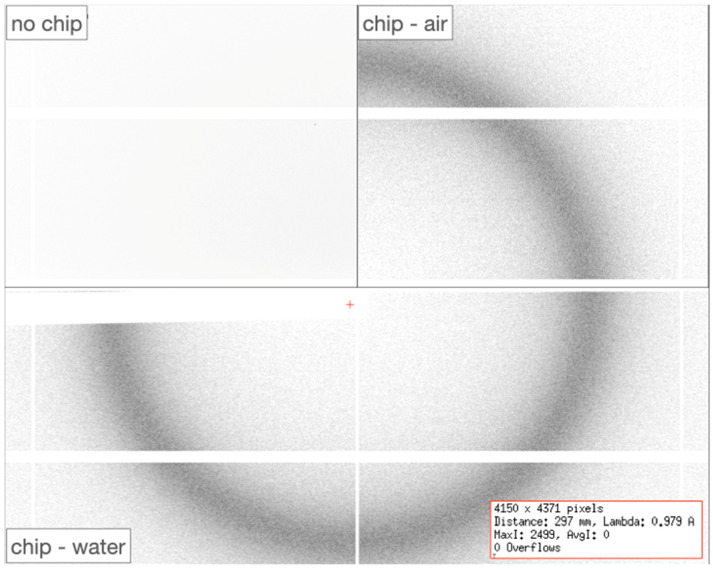
Diffusion background of the COC chip loaded with water (*chip-water)*, empty of any liquid (*chip-air*), in comparison with classical air scattering (*no chip*), as recorded at the beamline PROXIMA-1 of Synchrotron SOLEIL. The values of overflows, maximum intensity, and averaged intensity for the water-loaded chip are indicated in the bottom-right inset.

X-ray diffraction data were recorded on the PROXIMA-1 beamline at Synchrotron SOLEIL [29], equipped with an Eiger-16M (Dectris GmbH) and a three-rotation-axis SmarGon goniometer (SmarAct GmbH). For collecting diffraction data with reduced background noise, the direct beam stopper and the upstream beam pinhole were brought to ~7 mm from the sample. In this configuration, the rotation angle of the chip around the Omega-axis was limited to ±40° around the mounted position to avoid a potential collision with the hardware (Figure 2d). The nature and design of the MiD present minimum diffusing material to the incoming X-ray beam, resulting in close to noise-free diffraction experiments (Figure 3).

Following the procedure of small-wedged serial crystallography, a total of 21 datasets were collected on independent crystals, with an oscillation of 0.05° and exposure time of 0.01 s per image, at an X-ray energy of 12.67 keV. During collection, the crystals were illuminated with 3.8·10^10^ photons/sec distributed over 40 × 20 micron^2^, corresponding to an overall dose of approximately 1.8 kGy per crystal. Calculations for dose deposition were performed by *RADDOSE-3D* [30]. Data collection details are summarized in Table 1.

Data were processed with *XDS* [31] through the *autoProc* package [32], before being converted to MTZ format by *POINTLESS* [33], and scaled and merged by *AIMLESS* [34], as implemented within the *autoProc* procedure, taking as the resolution cutoff criteria CC_1/2_ >= 0.85 and I/sig(I) >= 2.5 in the outermost resolution shell. A total of 21 datasets were processed, scaled, and merged with no special attention provided to differentiate which data were better than the others. The crystals belonged to P4_3_2_1_2, with unit cell parameters 79.62 Å, 79.62 Å, 37.89 Å, and contained one molecule per asymmetric unit.

Structure factor amplitudes were obtained by *TRUNCATE* [35,36]. Rotational and translational functions were calculated and compared by *MOLREP* [37] using the PDB coordinates 1LYZ as a template model. The solved structure was then run through rounds of refinement with *BUSTER* [38] and manual model building using *Coot* [39]. The final model contained 129 residues at a resolution of 1.83 Å, with a final *R* factor and *R*_free_ values of 17% and 20%, respectively (Table 1). The final model was validated by *Molprobity* [40].

## 3. Results and Discussion

### 3.1. COC Devices for Diffusion-Based Crystallization

The overarching goal of the present study was to fabricate microfluidic devices designed for both diffusion-based crystallization of macromolecular crystallography and for in situ diffraction experiments at hard X-ray synchrotron sources. Crystallization by diffusion places in contact a precipitating agent with the protein sample in a convection-free environment such as presented by small microfluidic channels within microfluidic chips. Microfluidic devices for diffusion experiments have been reported in the past [1], introducing the concept of the fabrication of small channels and exploring the possibilities for diffraction experiments. The design of the channels and composition of the chips, however, have strongly influenced the quality of the recorded data and most likely affected the diffraction limit collected on the studied protein crystals.

**Figure 4:**
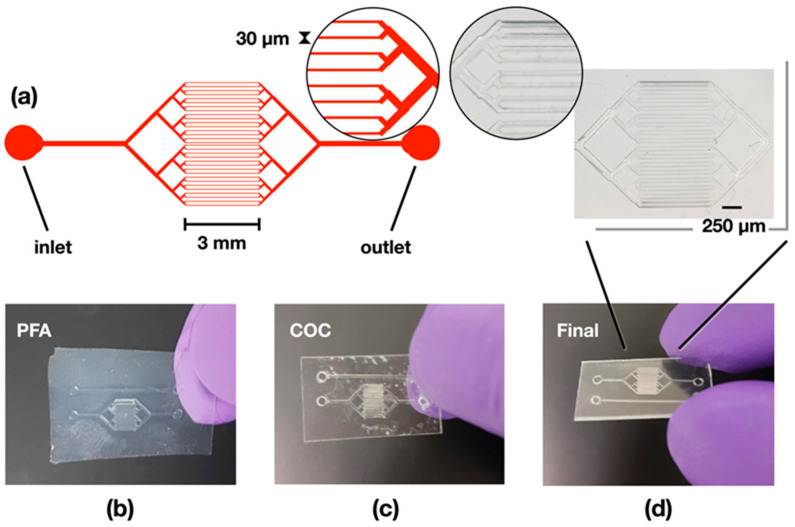
Stepwise fabrication of the microfluidic device setup. (**a**) Schematic representation of the MiD with the details of the dimensions for the channel cross-section (30 µm) and full length (3 mm) indicated in the diagram. The resulting parts of the manufacturing consist of (**b**) the PFA mold coming out of the PDMS, (**c**) the COC sheet with the PFA features, and (**d**) the final device after bonding the COC channels with the COC sheet. The inlet in (**d**) is a zoomed photo of the COC device, highlighting the details and small dimensions of the chip.

In the current production process, COC was favored over other materials to fabricate a diffusion-based crystallization device made of microfluidic channels with typical cross-section dimensions of 30 µm in width, 50 µm in height, and over a diffusion length of 3 mm (Figure 4a). The production of COC structures using replication techniques comfortably presents channels with resolutions of over 100 microns, but to our knowledge, structures with a controlled channel width in the order of 10–30 µm and a total thickness of 100–300 µm have not yet been achieved at the industrial level. The main difficulty in transferring such small features comes from the large amount of polymer that must be displaced during replication, combined with its high viscoelastic constraints.

To prepare COC devices with small cross-sections, the initial design of the channel was made into a photo mask from which a SU-8 master was fabricated by UV-photolithography. PDMS molds of these masters were used as stamps for hot embossing PFA imprints with the inverse channel structure, which, in turn, were used for hot embossing COC molds (Figure 1). Hot embossing refers here to a method whereby thermoplastic materials are heated above their glass transition temperature (T*_g_*), where they become viscous and moldable. The softness of PDMS when placed under higher pressures causes physical constraints and deformation, which can lead to replication errors if PDMS is used as the master. Consequently, the use of PFA (T*_g_*~90 °C, T*_m_* = 290 °C) was chosen as an intermediate stamp to transfer the micropattern onto COC (Figure 4b,c). Another advantage of using PFA lies in its elasticity and weak adhesion to substrates, hence allowing for an easier detachment from the brittle COC.

While COC is resistant to most polar solvents (e.g., ethanol and acetone), it is highly deformed by non-polar solvents such as cyclohexane or decahydronaphthalene hydrocarbons, which penetrate the COC polymer and cause swelling by spacing the polymeric chains. The COC sheets become sticky until all solvent has evaporated, making it possible to press together both surfaces to form a stable bond due to residual solvent penetrating the remaining non-sticky COC surface and jamming with the solvent-spaced polymer chains (Figure 4d).

### 3.2. Sample Injection, Crystal Formation, and Diffraction Studies

Before loading crystallization and sample solutions in the COC-type MiD, two dedicated inlets and outlets were added at the extremities of the chip (Figure 4a), on which PDMS blocks were plasma-bonded for easier tubing. The viscosity of the solutions strongly affects their injection in the microchannels, which may impair the crystallization process. The sample loading procedure was therefore validated by flow experiments visualized under digital microscopy after enclosing the COC chip in a light-weight chip holder for easier handling (Figure 2a). The sample is classically loaded at slow speeds to avoid the appearance of air bubbles in the MiD and to fill all the thirty-two channels in parallel. The pace of injection is adjusted by a mass flow controller that exploits pressure differences. In the current chip design, as little as ~1 µL of the precipitant solution was sufficient to occupy all the microchannels and reach the outlet, after which, approximately 0.5 µL of protein-containing solution can be injected using a pressure difference of 3 mBar on the mass flow controller. Loading two differently colored solutions to mimic the diffusion process shows a homogeneous gradient (Figure 2b).

When applying the crystallization and sample solutions, the inlet and outlet tubing need to be sealed to preserve the injected solutions from vaporing out. In the current experiments, Lysozyme crystals were confirmed to grow directly within the channels of the chip, reaching dimensions approximately the width of the channels 14 h after injections (Figure 2c). Moreover, the crystals appeared to be distributed at the center of the chip, confirming the efficacy of the injection process. After confirming the appearance of the crystals, the chip was mounted on the PROXIMA-1 beamline of Synchrotron SOLEIL for X-ray diffraction experiments. The central localization of the crystals had the unforeseen advantage of helping to quickly identify the positive crystallization hits for faster data collection procedures. The method of serial small-wedged data recording [41] was used to automate and simplify the procedure.

The structure of the Lysozyme was solved by the molecular replacement method applied to the recorded data merged from 21 independent crystals (Table 1). The MiD design and conception allow for minimizing the background noise originating from the interaction of the incoming X-rays with the material composing the chip (Online Resource 1). Consequently, the flux deposited on the in situ crystals could be lowered to 3.8·10^10^ photons/sec without affecting the quality of the diffraction experiments. By reducing the flux of the incoming X-ray beam, the deposited dose on each crystal could be lowered to 1.8 kGy, which is several orders of magnitude smaller than the Garman limit (30 MGy) [42] and, therefore, has the marked advantage of slowing down the impact of radiation damages on the crystals.

**Figure 5:**
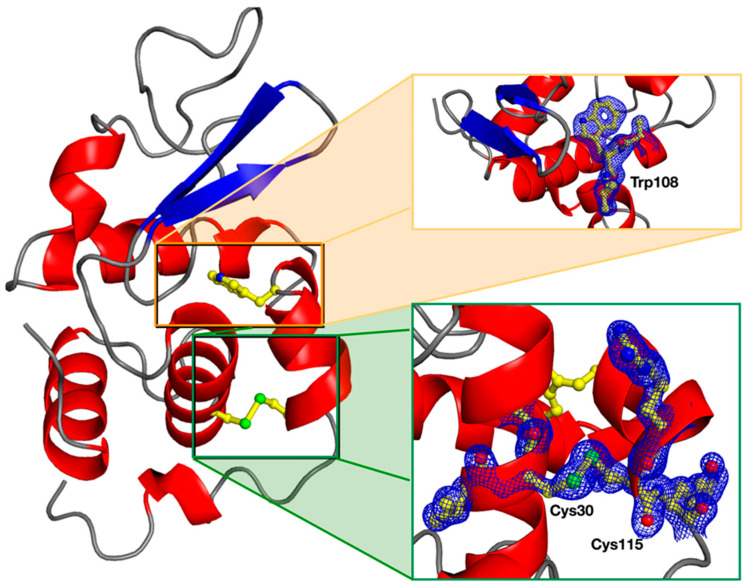
Overall structure of the Lysozyme refined at 1.83 Å resolution. The quality of the data can be seen in the two insets, showing the electron density around Trp108 and the disulfide bridge Cys30-Cys115.

The strategy to record small-wedged diffraction data was favored over faster still-image raster scanning methods to increase the partial completeness of each of the data on all crystals and, therefore, reduce the number of required crystals before obtaining the fully complete merged data. The final structure of the Lysozyme did not emphasize any markable conformational changes amenable to X-ray radiation damage, resulting in crystallographic statistics of good quality (Table 1). Overall, the 1.83 Å resolution electron density of the protein is clearly defined around all amino acids, including at the disulfide bridges, a classical trademark of radiation damage (Figure 5). The absence of noticeable damages in the electron density demonstrates the applicability of the MiD in combination with small-wedged serial crystallography for damage-less structural studies.

## 4. Conclusions

With the advances in technologies, the field of structural biology is in constant evolution and needs to adapt different methodologies in an integrated approach to combine the information coming from various sources before unraveling complex mechanisms. Microfluidics stands as a promising technique perfectly adapted to biological material, notably due to its malleable nature and infinite possibilities of design, which could be reshaped to fit any analysis method. In the current study, the design and fabrication process of a microfluidic device of small dimensions were shown to be perfectly suitable for diffusion-based crystallization and further in situ crystal analysis at X-ray diffraction sources. The performances of the chip in terms of miniaturization, background scattering, handling, and industrialization are much more favorable when compared to previously reported devices. The device consumes a small volume of sample at the phase of crystallization and takes advantage of small-wedged in situ serial crystallography for minimizing the amount of data to acquire for a complete dataset. The use of COC as the source material of the chip, its small dimensional design, and its transparency to energies ranging from infra-red, deep ultra-violet, to X-rays are all arguments that push toward the exploitation of these devices for complementary biological analysis techniques. The current work provides yet further emphasis on the fantastic potential and adaptability of microfluidics when combined with the experimental stations implemented at synchrotron radiation sources. Future COC MiD developments at our facilities will encompass the incorporation of additional inlets for diffusion experiments of the newly formed crystals with potential drug targets. By functionalizing the chip with such optional diffusion routes, fragment-based drug discovery and dynamical studies will become possible.

## Figures and Tables

**Table 1:** Data and refinement statistics. Values in parentheses correspond to the highest resolution shell.

DATA COLLECTION	
Space group	P4_3_2_1_2
Unit cell parameters (Å, °)	*a* = *b* = 79.61, *c* = 37.88, *α* = *β* = *γ* = 90
Resolution (Å)	56.28–1.83 (1.86–1.83)
No. of observed reflections	306,732 (4445)
No. of unique reflections	11,146 (525)
Completeness (%)	100 (99.4)
*R*_merge_	0.111 (0.578)
*R*_meas_	0.113 (0.614)
*R*_pim_	0.021 (0.2)
〈*I*/σ(*I*)〉	22.2 (2.8)
CC_1/2_	0.999 (0.884)
Multiplicity	27.5 (8.5)
Wilson *B* factor (Å^2^)	28.68
**REFINEMENT**	
*R*_free_	0.19
*R*_work_	0.17
r.m.s.d., bond lengths/angles (Å, °)	0.008/0.92
Ramachandran (favored/allowed, %)	99.21/0.79
Average *B* factor (Å^2^)	
Overall	29.18
For protein residues	27.73
For water	44.52

## Data Availability

Not applicable.
